# Influence of Social and Behavioural Characteristics of Users on Their Evaluation of Subjective Loudness and Acoustic Comfort in Shopping Malls

**DOI:** 10.1371/journal.pone.0054497

**Published:** 2013-01-15

**Authors:** Qi Meng, Jian Kang

**Affiliations:** 1 School of Architecture, Harbin Institute of Technology, Harbin, China; 2 School of Architecture, University of Sheffield, Sheffield, United Kingdom; Universite Paris Sud, France

## Abstract

A large-scale subjective survey was conducted in six shopping malls in Harbin City, China, to determine the influence of social and behavioural characteristics of users on their evaluation of subjective loudness and acoustic comfort. The analysis of social characteristics shows that evaluation of subjective loudness is influenced by income and occupation, with correlation coefficients or contingency coefficients of 0.10 to 0.40 (*p*<0.05 or *p*<0.01). Meanwhile, evaluation of acoustic comfort evaluation is influenced by income, education level, and occupation, with correlation coefficients or contingency coefficients of 0.10 to 0.60 (*p*<0.05 or *p*<0.01). The effect of gender and age on evaluation of subjective loudness and acoustic comfort is statistically insignificant. The effects of occupation are mainly caused by the differences in income and education level, in which the effects of income are greater than that of education level. In terms of behavioural characteristics, evaluation of subjective loudness is influenced by the reason for visit, frequency of visit, and length of stay, with correlation coefficients or contingency coefficients of 0.10 to 0.40 (*p*<0.05 or *p*<0.01). Evaluation of acoustic comfort is influenced by the reason for visit to the site, the frequency of visit, length of stay, and also season of visit, with correlation coefficients of 0.10 to 0.30 (*p*<0.05 or *p*<0.01). In particular, users who are waiting for someone show lower evaluation of acoustic comfort, whereas users who go to shopping malls more than once a month show higher evaluation of acoustic comfort. On the contrary, the influence of the period of visit and the accompanying persons are found insignificant.

## Introduction

There are about 20,000 shopping malls in China [1]. Harbin City, for example, has 30 shopping malls, while 15 more will be constructed before 2020 [2]. The evaluation of acoustic environment in such spaces has been paid increasing attention by users, researchers and governmental organisations [3]–[4]. While some studies have been carried out in terms of acoustic environment in shopping malls, the main focus has been on noise control [5] and also, only limited types of shopping mall, such as underground shopping centres, have been considered [6]–[7].

Previous studies suggested that the sound environment evaluation of a space depends strongly on the social characteristics of the users, such as gender, age, income, occupation, and education, as well as their behavioural characteristics [8]–[24]. Mehrabian [12] indicated a slight tendency for women to be more sensitive to sound than men, and evidence suggests that females generally have a higher arousal level than males [13]. In terms of age influence, Yang and Kang [14] found in their research on urban open public spaces that users are more favourable of, or tolerant towards sounds relating to nature with the increase of age. With regards to the education factor, Yu and Kang [15]–[17] indicated that the correlation coefficients for natural sounds are predominantly negative, suggesting that people tend to prefer natural sounds more with the increase in education level. For human sounds, mixed positive and negative correlation coefficients are found. Kang [18] indicated that the behavioural characteristics of users may also play an important role, and sound quality of an urban area may depend on how long people have been living there [19]. Similarly, Bull [20] found that people with stereos may have different sound evaluation from others. Bertoni et al. [21] also stated that sound experience is important, but Job et al. [22] obtained contradictory results. Della Crociata et al. [23]–[24] indicated that users' acoustic satisfaction is highly correlated with perceived acoustic intensity and is influenced by sources of acoustic annoyance. Cultural factors play an important role as well; hence, the effect of social and behavioural factors vary significantly between different countries [25]–[26].

Consequently, the present study is conducted to examine the influence of social and behavioural factors on evaluation of subjective loudness and acoustic comfort in different types of shopping mall, based on a series of subjective surveys. The social factors considered include gender, age, income, occupation, and education; whereas the behavioural factors include the reason for visit, frequency of visit, length of stay, period of visit, and number of accompanying partners.

## Methods

### Ethics statement

This study was approved by the Degree Committee of the School of Architecture, Harbin Institute of Technology (this governing body is equipped with an ethical review board). In the questionnaire the names of individual participants were not included, and participants provided their written informed consent to participate in this study. Participants aged 17 were accompanied by their parents.

### Survey sites

The field study was conducted through a questionnaire survey at selected case study sites in Harbin, China. Harbin, the capital city of Heilongjiang province, is a typical large city in China, characterised by a continental climate, with four highly distinguishable seasons. The population is about 10 million, and its economic development is at medium level in China [2].

The 30 existing shopping malls in Harbin were first divided into two groups based on their space types, that was, 12 were considered as single-space type, where the space was generally of a single volume and it was seen as one room on the plan, and 18 were multiple–space type, where the space consisted of multiple volumes and on the plan there were more than one linked rooms. This division according to space types was based on previous studies stating that space types are an important factor affecting the evaluation of acoustics in indoor spaces because reverberation time (RT) and sound pressure level (SPL) may vary in different space types [18], [27]. Three shopping malls from each space type were randomly selected as final samples. The six case study shopping malls were Qiu Lin, Tong Ji, Man Ha Dun, Suo Fei Ya, Jin An, and Hui Zhan, among which Qiu Lin, Man Ha Dun and Hui Zhan were single-space type, the others were multiple–space type. Details about these shopping malls are presented in Table 1. It is noted that Hui Zhan is a typical underground shopping mall, with which comparison could be made between under- and above-ground shopping malls.

**Table 1:** Basic information of the survey sites.

Sites	Size (m^2^)	Sound sources	NI	Aver. SPL (dBA)	Aver. SL	Aver. AC
Qiu Lin	31000	Background music, music from shops, PA system, footsteps, surrounding speech, air-conditioning	372	71.31	3.36	3.08
Tong Ji	10000	Background music, vendors' shouting, music from shops, sounds from toys, surrounding speech, footsteps, air-conditioning	188	73.28	3.52	2.73
Man Ha Dun	28700	Background music, vendors' shouting, music from shops, sounds from toys, surrounding speech, footsteps, air-conditioning	302	71.43	3.48	2.96
Suo Fei Ya	32000	Background music, music from shops, PA system, footsteps, surrounding speech, air-conditioning	285	70.80	3.32	2.80
Jin An	45000	Background music, music from shops, PA system, footsteps, surrounding speech, air-conditioning	297	68.31	3.20	3.41
Hui Zhan	30000	Background music, vendors' shouting, music from shops, PA system, footsteps, surrounding speech, air-conditioning, water	690	69.42	3.30	3.27

The basic information includes size, sound sources, the number of interviews conducted, average SPL, average subjective loudness (1, very quiet; 2, quiet; 3, neither quiet nor loud; 4, loud; and 5, very loud) and acoustic comfort (1, very uncomfortable; 2, uncomfortable; 3, neither comfortable nor uncomfortable; 4, comfortable; and 5, very comfortable). ‘NI’ means number of interviews, ‘Aver. SL’ means average evaluation of subjective loudness, and ‘Aver.AC’ means average evaluation of acoustic comfort.

### Questionnaire survey

Around 100 to 700 valid questionnaires were obtained in every shopping mall from autumn 2011 to summer 2012. In total, 2,134 questionnaires were received. Previous studies suggested that 100 valid questionnaires are appropriate to evaluate the acoustics of a particular place [17]–[18], whereas to examine the effects of users' social or behavioural factors a considerably larger number of questionnaires would be needed [18]. The interviewees in the case study sites were randomly selected, and based on the initial data analysis, their educational and social backgrounds, as well as on-site behaviours (such as waiting for someone, walking around, shopping or passing by), were considered as representatives [22]–[26]. Generally 10–15 fixed survey positions were used, which were equally distributed at every survey site, and more than 20 meters from each other [18].

The questionnaire included four parts, according to the framework suggested by Kang [18], considering user, space, sound, and environment. The first part was about a user's basic information in terms of social characteristics (such as gender, age, income, education level, and occupation) and behavioural characteristics (such as what they are doing, frequency of visit, time of visit, and accompanying persons). The second part was on the evaluation of space, especially the evaluation of reverberation and space perception. The third part was about sound sources and the evaluation of loudness. The final part was the evaluation of other environmental factors, such as temperature, humidity, and lighting. The questionnaire was introduced as an enquiry on general environmental conditions, instead of concentrating solely on acoustic environment, to avoid possible bias towards the acoustic aspect [28]. The questionnaire's reliability and validity were tested before the actual field surveys [29].

A five-point bipolar category scale was used in the questionnaire. Evaluation of subjective loudness was divided into five levels: 1- very quiet; 2- quiet; 3- neither quiet nor loud; 4- loud; and 5- very loud. Evaluation of acoustic comfort was also divided into five levels: 1- very uncomfortable; 2- uncomfortable; 3- neither comfortable nor uncomfortable; 4- comfortable; and 5- very comfortable. For other questions and scales in the questionnaire, more details can be found from [29].

The surveys covered four seasons because previous studies in urban open public spaces indicated that seasons may affect users' evaluation of acoustics [17]. Moreover, the surveys were conducted at various times of day, from morning to afternoon [14], [18]. Three time periods were considered, namely, 09:00 to 11:59 (morning), 12:00 to 14:59 (midday), and 15:00 to 17:59 (afternoon).

### Objective measurements

The sound level measurement was conducted immediately after each questionnaire interview, and the microphone of the sound level meter was positioned near the location of questionnaire interview and more than 1 m away from any reflective surfaces and 1.2 m to 1.5 m [18] above the floor to avoid the effect of sound reflections. The sound level meter was set into slow-mode, and reading was taken every 3 s to 5 s. A total of 100 measurement data were obtained in each survey position, and the corresponding A-weighted equivalent continuous sound level, LAeq, was derived [30]–[32]. In other words, after each interview a 300–500 s measurement was made. Simultaneously, other environmental factors, such as air temperature, relative humidity, and lighting were also measured at the survey positions corresponding to every sound level measurement [33]–[34], although in this paper, due to the limitation of space, these data are not included and analysed.

### Data analysis

SPSS 15.0 was used to establish a database with all the subjective and objective results [35]. The data were analysed using the following: Chi-square correlations (two-tailed) for factors with three or more categories of ranked variables; Chi-square contingency correlations (two-tailed) for factors with three or more categories for categorical variables; and mean differences t-test (two-tailed) for factors with two categories. Both linear and nonlinear correlations were considered [36].

## Results

### Social characteristics

#### Gender

Although previous studies suggested that the effect of gender on sound annoyance evaluations is generally insignificant [37]–[38], they are more focused on urban open spaces. In this study, the mean difference in evaluation of subjective loudness and acoustic comfort was determined between males and females of every survey site, as shown in Table 2. It is interesting to note that in shopping malls, again, no significant (*p*>0.1) difference was observed between males and females.

**Table 2:** Relationships between social characteristics and evaluation of subjective loudness, as well as acoustic comfort.

	Gender		Age groups		Education level		Income		Occupation	
Survey sites	SL	AC	SL	AC	SL	AC	SL	AC	SL	AC
Qiu Lin	0.08	−0.17	0.05	−0.02	0.02	−0.33^**^	0.22^**^	−0.36^**^	0.21^**^	0.17^**^
Tong Ji	0.09	−0.08	0.11	−0.01	0.04	−0.41^**^	0.14^*^	−0.42^**^	0.16^*^	0.22^**^
Man Ha Dun	0.14	−0.08	0.03	0.00	0.05	−0.38^**^	0.15^*^	−0.40^**^	0.12^*^	0.20^**^
Suo Fei Ya	0.06	0.00	0.06	0.07	0.03	−0.36^**^	0.26^**^	−0.37^**^	0.25^**^	0.17^*^
Jin An	0.04	−0.10	0.04	−0.10	0.06	−0.46^**^	0.32^**^	−0.51^**^	0.18^**^	0.21^**^
Hui Zhan	0.01	−0.07	−0.01	−0.05	0.04	−0.45^**^	0.30^**^	−0.47^**^	0.13^*^	0.16^*^

The table includes mean difference between males and females, chi-square test correlation coefficients forage groups, income, education level, and chi-square test contingency coefficients for occupation, where the significance levels are also shown, with ** indicating p<0.01, and *indicating p<0.05. SL represents evaluation of subjective loudness, and AC represents evaluation of acoustic comfort.

#### Age

Previous studies indicated that different age groups may have different evaluations of the sound environment and sound preference, possibly because of their long-term experience [19], [21], [39]–[40]. In this study, the users' age were divided into 7 groups, namely, < = 17, 18 to 24, 25 to 34, 35 to 44, 45 to 54, 55 to 64, and > = 65 [17]. The relationships between age groups and evaluation of subjective loudness and acoustic comfort are shown in Table 2. It can be seen that there is no significant difference (*p*>0.1) among the age groups in terms of evaluation of subjective loudness or acoustic comfort. Table 3 further examines whether there are some tendencies that certain age groups would rate the evaluation of subjective loudness and acoustic comfort more extremely. It seems that no such tendencies are observed.

**Table 3:** Differences among age groups in terms of evaluation of subjective loudness and acoustic comfort.

Survey sites	Very loud	Very quiet	Very comfortable	Very uncomfortable
Qiu Lin	< = 17	35–44	> = 65	25–34
Tong Ji	25–34	45–54	55–64	< = 17
Man Ha Dun	< = 17	25–34	18–24	55–64
Suo Fei Ya	45–54	18–24	18–24	35–44
Jin An	35–44	55–64	< = 17	45–54
Hui Zhan	> = 65	< = 17	18–24	35–44

#### Education level

In China, educational level can be divided into 6 groups, namely, (1) non literate, (2) primary school, (3) junior middle school, (4) senior middle school, special school or technical school, (5) college graduates, and (6) graduate or higher [34]. In this study, only three people were at the level of non literate, so only the five other levels were considered. The relationships between education level and evaluation of subjective loudness, as well as acoustic comfort, are shown in Table 2. No significant (*p*>0.1) difference was found between education level and subjective loudness, but the correlation between education level and acoustic comfort was from −0.30 to −0.50 (*p*<0.01). In other words, the higher the users' education level is, the lower the acoustic comfort is.

#### Income

The users' income level was divided into 6 groups, namely, < = 1000, 1001–2000, 2001–3000, 3001–4000, 4001–5000, and > = 5001 Yuan (1 Yuan ≈0.15 US dollar) per month [36]. The correlations between users' income and evaluation of subjective loudness, as well as acoustic comfort, as shown in Table 2, where the income is measured by US dollar, are from 0.10 to 0.40 (*p*<0.01), and from −0.30 to −0.60 (*p*<0.01), respectively. In other words, the higher the users' income is, the higher the users' evaluation of subjective loudness is, but the lower the evaluation of acoustic comfort is.

**Figure 1:**
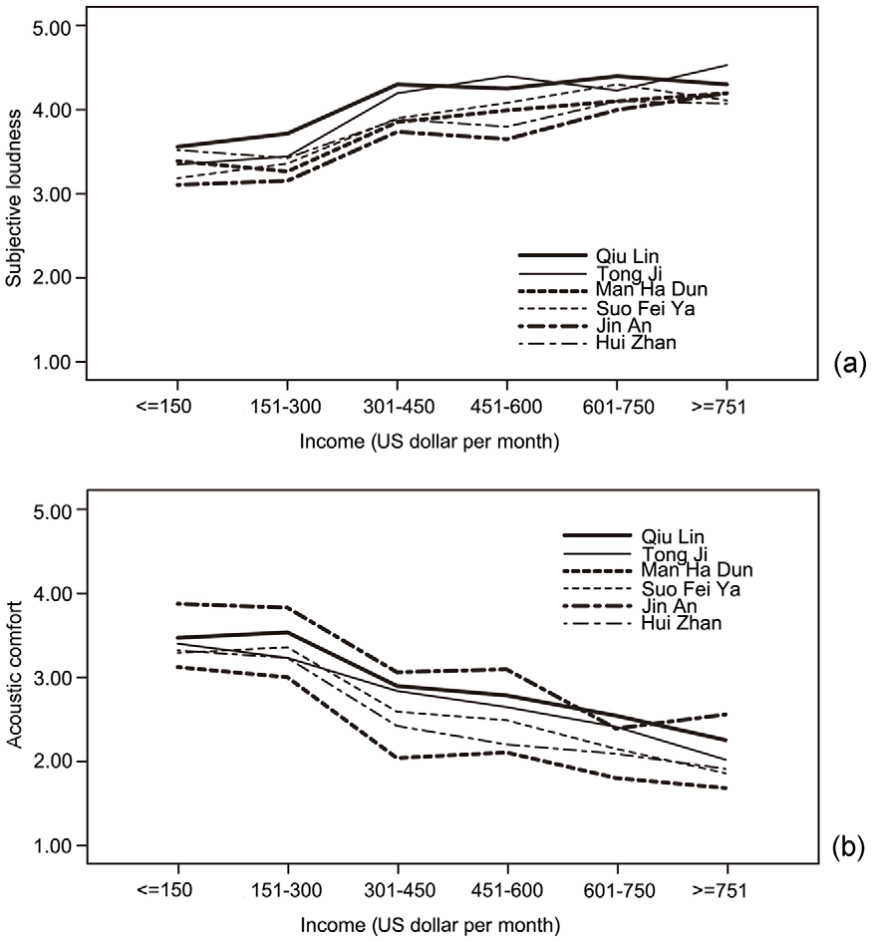
Relationships between users ' **average income and their evaluation of acoustics.** (a) Between income and evaluation of subjective loudness; (b) Between income and evaluation of acoustic comfort.

The relationships between users' income and evaluation of subjective loudness, as well as acoustic comfort, are also illustrated in Figure 1. It is interesting to note that, both evaluation of subjective loudness and acoustic comfort change significantly when the income level changes from 301 to 450 US dollar per month, which is approximately the average income of people in Harbin, namely 2,450 Yuan per month (≈367 US dollar) in 2011 and 2,518 Yuan per month (≈378 US dollar) in 2012 [41]–[42]. In other words, it seems that in terms of evaluation of subjective loudness and acoustic comfort, there is a significant difference between people with “lower than average income (<2000 Yuan)” and “higher than average income (>3000 Yuan)”, with a mean difference of 0.31 to 0.52 on subjective loudness (*p*<0.01), and of 0.49 to 0.76 on acoustic comfort (*p*<0.01).

#### Occupations

The users' occupations were divided into 11 groups, namely, farmer, soldier, worker, service worker, technical worker, teacher, officer, student, self-employed individual, housewife, retiree, and unemployed [43]. The relationships between users' occupations and evaluation of subjective loudness and acoustic comfort are shown in Table 2, showing Chi-square contingency correlation of 0.10 to 0.30 (*p*<0.05) and 0.10 to 0.30 (*p*<0.05), respectively. In other words, with different occupations, evaluation of subjective loudness and acoustic comfort also differ.

The occupations that corresponded to extreme evaluation of subjective loudness and acoustic comfort are shown in Table 4. It can be seen that farmers tend to select ‘very loud’ for evaluation of subjective loudness, and their evaluation of subjective loudness is 0.06 to 0.12 higher than that of the other occupations (*p*<0.01) in four out of six survey sites, namely Qiu Lin, Tong Ji, Man Ha Dun and Hui Zhan. Given that farmers usually live in a relatively quiet environment, they may feel nosier when they go to shopping malls in big cities. However, they may not necessarily feel the acoustic environment is unconformable, as can also be seen in Table 4. In the questionnaire, one farmer stated that “I would like to be surrounded by noisy crowd.” It is also interesting to note that soldiers may have higher evaluation of acoustic comfort than others, with a mean difference of 0.02 to 0.07 (*p*<0.05) in four survey sites, namely Qiu Lin, Suo Fei Ya, Jin An, and Hui Zhan.

**Table 4:** Differences among occupations in terms of evaluation of subjective loudness and acoustic comfort.

Survey sites	Very loud	Very quiet	Very comfortable	Very uncomfortable
Qiu Lin	Farmer	Worker	Soldier	Teacher
Tong Ji	Farmer	Student	Retire	Farmer
Man Ha Dun	Farmer	Worker	Self employed individual	Farmer
Suo Fei Ya	Retire	Officer	Soldier	Retire
Jin An	Technical man	Soldier	Soldier	Student
Hui Zhan	Farmer	Soldier	Soldier	Retire

### Behavioural characteristics

#### Reason for visit

Users generally have four purposes for coming to the survey sites, namely, shopping, walking, passing by, and waiting for someone. The relationships between the reason for visit and evaluation of subjective loudness and acoustic comfort are shown in Table 5. The contingency correlation between the reason for visit and evaluation of subjective loudness and acoustic comfort was from 0.10 to 0.20 (*p*<0.05) and from 0.10 to 0.30 (*p*<0.05), respectively. In other words, the users' evaluation of acoustic evaluation may be influenced by their different reasons for visit to the shopping malls.

**Table 5:** Relationship between behavioural characteristics and evaluation of subjective loudness, as well as acoustic comfort.

	Reason for visit		Frequency of coming		Season		Period of visit		Length of stay		Accompanying persons	
Survey sites	SL	AC	SL	AC	SL	AC	SL	AC	SL	AC	SL	AC
Qiu Lin	0.13^**^	0.21^**^	−0.26^**^	0.22^**^	0.03	0.21^**^	0.04	0.02	−0.24^**^	0.21^**^	0.02	0.06
Tong Ji	0.17^**^	0.16^*^	−0.30^**^	0.19^**^	0.01	0.16^*^	0.06	0.00	−0.16^**^	0.26^**^	0.06	0.02
Man Ha Dun	0.16^*^	0.14^*^	−0.23^**^	0.24^**^	0.07	0.12^*^	0.03	0.03	−0.33^**^	0.13^*^	0.01	0.07
Suo Fei Ya	0.18^**^	0.19^*^	−0.33^**^	0.18^**^	0.05	0.20^**^	0.07	0.04	−0.27^**^	0.20^**^	0.04	0.01
Jin An	0.11^*^	0.24^**^	−0.27^**^	0.26^**^	0.09	0.17^*^	0.02	0.04	−0.22^**^	0.18^**^	0.07	0.01
Hui Zhan	0.18^**^	0.09	−0.18^**^	0.13^**^	0.17	0.18^*^	0.03	0.08	−0.14^**^	−0.01	0.01	0.03

The table includes mean difference between persons with partners and without, chi-square test correlation coefficients for frequency of coming, income, education level, and chi-square test contingency coefficients for reason for visit, where the significance levels are also shown, with ** indicating p<0.01, and * indicating p<0.05. SL represents evaluation of subjective loudness, and AC represents evaluation of acoustic comfort.

**Figure 2:**
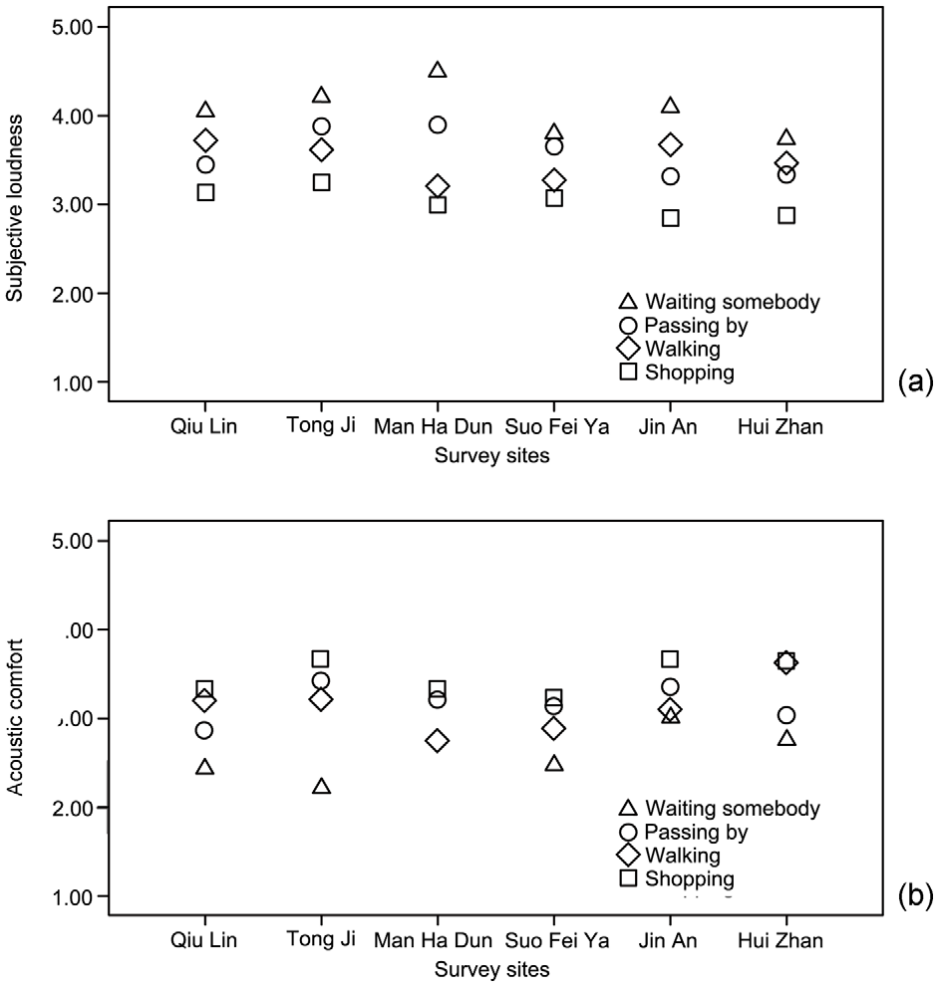
Effects of users' reason for visit on their evaluation of acoustics. (a) Evaluation of subjective loudness; (b) Evaluation of acoustic comfort.

The average evaluation of subjective loudness and acoustic comfort with different reasons for visit in all survey sites are shown in Figure 2. It can be seen that the users who came for shopping generally have lower evaluation of subjective loudness but higher evaluation of acoustic comfort, with a mean difference of 0.11 to 0.45 (*p*<0.01) and 0.15 to 0.58 (*p*<0.01) respectively, compared with other reasons. Conversely, the users who came to wait for someone generally have higher evaluation of subjective loudness, but lower evaluation of acoustic comfort, with a mean difference of 0.13 to 0.51 (*p*<0.01) and 0.09 to 0.49 (*p*<0.05 or *p*<0.01), respectively, compared with other purposes. This is likely because the users who are concentrating on shopping may pay decreased attention to the acoustic environment; whereas those who are waiting for someone could have increased attention.

#### Frequency of visit

The users' frequency of visit to a certain place and its influence on their evaluation of subjective loudness or acoustic comfort has been mentioned in previous studies [25], [37]. In this study, the users' frequency of visit to shopping malls was divided into 5 categories, namely, first time, rarely (at least once a year), sometimes (at least once a month), often (at least once a week), and frequently (more than three times a week). The relationships between the frequency of visit and evaluation of subjective loudness and acoustic comfort are shown in Table 5. It can be seen that both evaluation of subjective loudness and acoustic comfort are influenced by the frequency of visit, with correlation of −0.10 to −0.40 (*p*<0.01) in evaluation of subjective loudness, and 0.10 to 0.30 in evaluation of acoustic comfort, respectively. In other words, with the increase in the frequency of visit, the users' evaluation of subjective loudness is lower, but the evaluation of acoustic comfort is higher. This suggests that when users are more familiar with the environment of a certain space, such as shopping malls, they would have a better acoustic evaluation.

**Figure 3:**
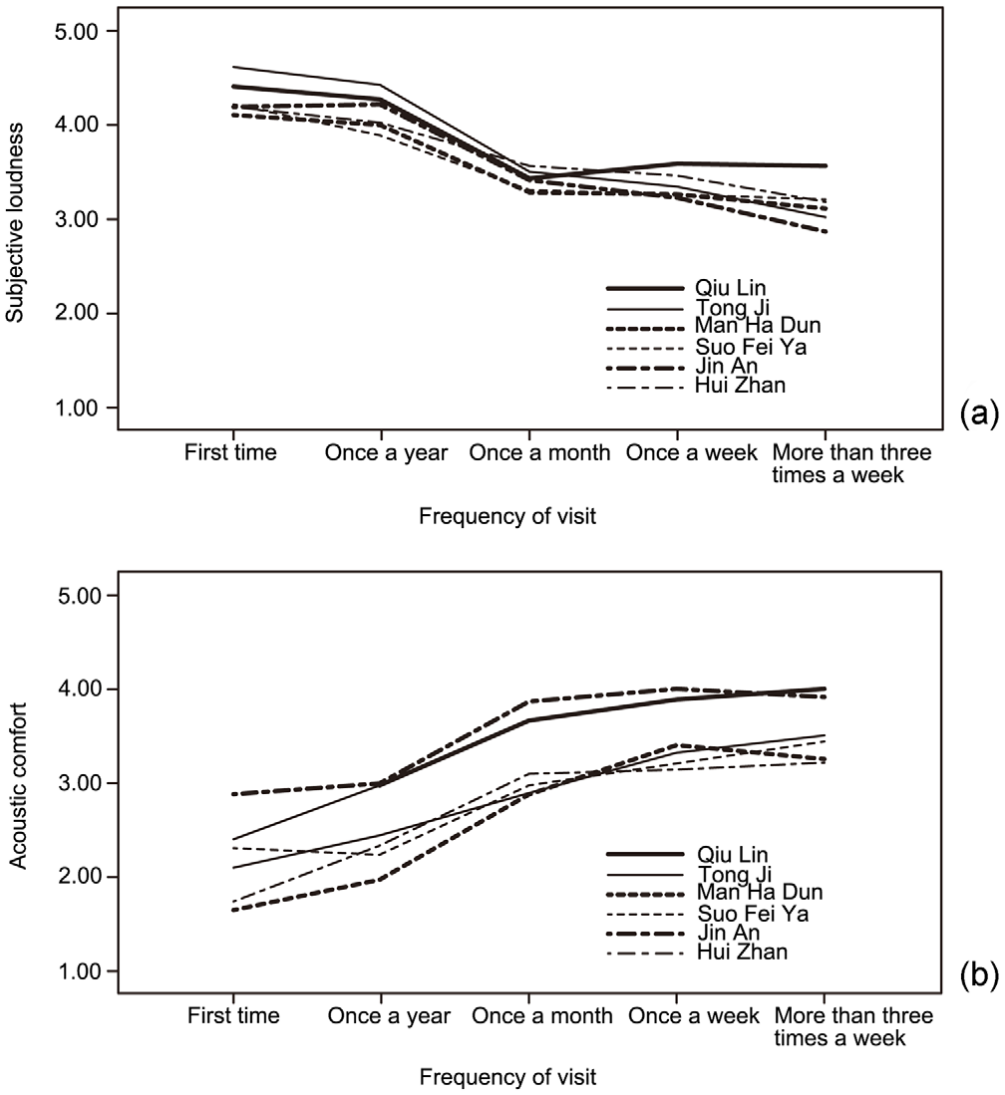
Relationships between users' frequency of visit and their evaluation of acoustics. (a) Between frequency of visit and evaluation of subjective loudness; (b) Between frequency of visit and evaluation of acoustic comfort.

Figure 3 presents the average evaluation of subjective loudness and acoustic comfort in all survey sites with increasing frequency of visit. It is interesting to note that in terms of evaluation of subjective loudness, there is a significant change from “once a year” to “once a month” (*p*<0.01), by0.52 to 0.81 in different sites. Meanwhile, the change in acoustic comfort is also significant, by 0.58 to 1.17 (*p*<0.01).

#### Time of visit

In this study, “time” was considered at three levels, namely seasons (from autumn to summer), period of visit (morning, midday, and afternoon); and length of stay (less than 1 hour, 1 to 2 hours, 2 to 3 hours, 3 to 4 hours, and more than 4 hours).

- *Seasons*. The relationships between seasons and evaluation of subjective loudness and acoustic comfort are shown in Table 5. No significant difference (*p*>0.1) was observed between seasons in terms of evaluation of subjective loudness, but significant differences were noted between seasons in terms of evaluation of acoustic comfort, with contingency correlation coefficients of 0.10 to 0.20 (*p*<0.05). This is perhaps because the evaluation of acoustic comfort is more related to the evaluation of other aspects of physical comfort, such as the heat and humidity conditions, and users in summer and winter usually give better evaluation for the indoor environment due to more comfortable thermal conditions in shopping malls [44], as one customer stated: “I prefer to shop in the malls in summer because they have air conditioning”.
- *Period of visit*. No significant (*p*>0.1) correlation was found between the period of visit and evaluation of subjective loudness, and also between the period of visit and acoustic comfort (see Table 5). While in urban open spaces the period of visit was found to be influential on the soundscape evaluation [45]–[46], perhaps because better lighting evaluation may bring better acoustic evaluation, in indoor shopping malls this may not be the case.
- *Length of stay*. The length of stay in a certain place is considered to have some influence on evaluation of acoustic comfort, as indicated in several previous studies, although those studies concerned evaluation of subjective loudness rather than acoustic comfort [47]–[48]. The relationships between the length of stay and evaluation of subjective loudness and acoustic comfort are given in Table 5, where it can be seen that the correlation coefficients are −0.10 to −0.40 (*p*<0.01), and 0.10 to 0.30 (*p*<0.05), respectively. In other words, the users will have lower evaluation of subjective loudness and higher acoustic comfort when their length of stay is longer.

**Figure 4:**
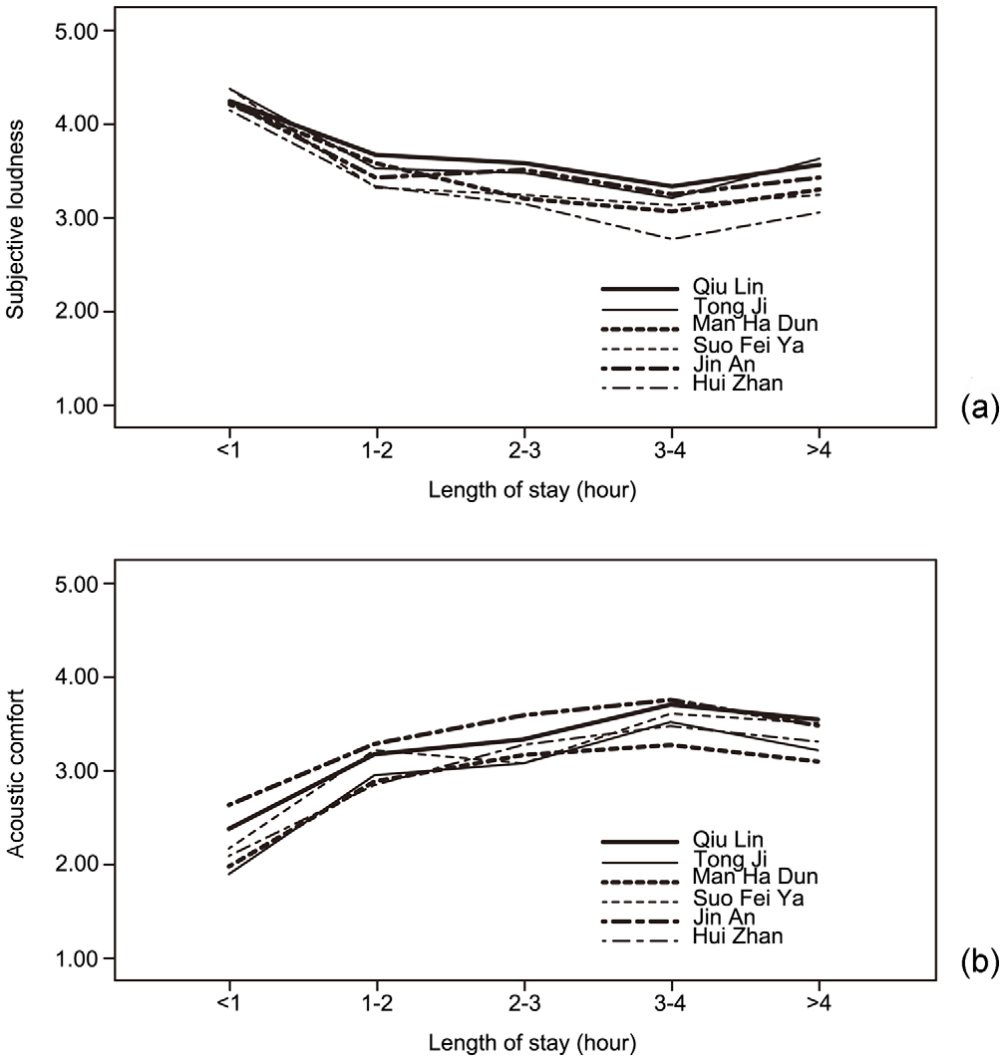
Relationshipsbetween users' length of stay and their evaluation of acoustics. (a) Between length of stay and evaluation of subjective loudness; (b) Between length of stay and evaluation of acoustic comfort.

The relationships between the length of stay and evaluation of subjective loudness and acoustic comfort are also shown in Figure 4. It can be seen that in terms of evaluation of subjective loudness there is a significant decrease from “less than 1 hour” to “1–2 hours”, with a mean difference of 0.52 to 0.98 (*p*<0.01) at different sites, whereas in terms of evaluation of acoustic comfort there is a significant increase, with a mean difference of 0.37 to 0.86 (*p*<0.01). Such changes in evaluation of subjective loudness and acoustic comfort continue with increasing length of stay until ‘3–4 hours’, although the rate of change is much slower. Conversely, when the length of stay is greater than 4 hours, evaluation of subjective loudness will increase and the acoustic comfort will decrease. Similar results were also obtained in a previous study [27].

#### Accompanying persons

Previous studies in urban open public spaces suggested that the acoustic evaluation might be different with accompanying persons [17]–[18], [29], [49]. In this study, however, no significant difference was obtained (*p*>0.1) between the users with accompanying persons and without, as shown in Table 5, in terms of evaluation of subjective loudness as well as acoustic comfort. A possible reason for this difference is that the users staying outdoors may pay more attention to their partners, compared to indoor spaces like shopping malls.

## Discussion

Previous studies noted that there are interrelationships between social characteristics [50]–[51]. This is also the case in this study, in terms of income, education level, and occupation, as shown in Table 6.

**Table 6:** Relationships among income, education level, and occupation.

Survey sites	Income and education level	Occupation and income	Occupation and education level
Qiu Lin	0.43^**^	0.30^**^	0.26^**^
Tong Ji	0.50^**^	0.35^**^	0.32^**^
Man Ha Dun	0.38^**^	0.28^**^	0.33^**^
Suo Fei Ya	0.32^**^	0.26^**^	0.24^**^
Jin An	0.42^**^	0.32^**^	0.36^**^
Hui Zhan	0.60^**^	0.37^**^	0.30^**^

The table shows chi-square test correlation coefficients between income and education level, and chi-square test contingency coefficients between occupation and income as well as education level, where the significance levels (2-tailed) are also shown, with ** indicating p<0.01, and * indicating p<0.05.

Relationships between occupation and evaluation of acoustic comfort are generally insignificant when income or education level is in a certain range. For example, considering the income “between 151 and 300 US dollar” or the education level of “graduate or higher,” the results in Table 7 indicate that generally there is no significant correlation between occupation and evaluation of acoustic comfort. Meanwhile, for a given occupation, such as “worker”, there are significant correlations between income and evaluation of acoustic comfort, with correlation coefficients of −0.30 to −0.60, as well as between education level and evaluation of acoustic comfort, with correlations of −0.30 to −0.50, as shown in Table 8.

**Table 7:** Relationships between occupation and evaluation of acoustic comfort.

Survey sites	Occupation and acoustic comfort with come fixed	Occupation and acoustic comfort with education level fixed
Qiu Lin	0.08	0.11
Tong Ji	0.10	0.05
Man Ha Dun	0.05	0.10
Suo Fei Ya	0.12	0.08
Jin An	0.04	0.13^*^
Hui Zhan	0.16	0.11

The table shows chi-square test contingency coefficients between occupation and evaluation of acoustic comfort, when income or education is fixed at a level, namely income is from 151 to 300 US dollar, education level is graduate or higher, where the significance levels (2-tailed) are also shown, with ** indicating p<0.01, and *indicating p<0.05.

**Table 8:** Relationships between users' evaluation of acoustic comfort and income, as well as education level.

Survey sites	Income	Education level
Qiu Lin	−0.40^**^	−0.36^**^
Tong Ji	−0.45^**^	−0.42^**^
Man Ha Dun	−0.41^**^	−0.40^**^
Suo Fei Ya	−0.39^**^	−0.38^**^
Jin An	−0.53^**^	−0.50^**^
Hui Zhan	−0.49^**^	−0.47^**^

The table shows chi-square test correlation coefficients between income or education level and evaluation of acoustic comfort, when occupation is fixed as worker, where the significance levels (2-tailed) are also shown, with ** indicating p<0.01, and * indicating p<0.05.

A multiple regression analysis [52] was then carried out to identify whether income or education level is more important for the evaluation of acoustic comfort. Table 9 shows that the *R_adj_^2^*, ranged 0.263 to 0.377, is significant (*p*<0.001) in all survey sites. Income is found to be generally more important than education level with regards to influencing the evaluation of acoustic comfort because it has higher absolute values, from 0.047 to 0.233, of standardised coefficient in all survey sites.

**Table 9:** The effect of users' income and education on evaluation of acoustic comfort.

Survey sites	*R*_adj_^2^	Factor	Standardised coefficient
Qiu Lin	0.312	Income	−0.332**
		Education level	−0.285**
Tong Ji	0.287	Income	−0.410**
		Education level	−0.207**
Man Ha Dun	0.365	Income	−0.338**
		Education level	−0.109**
Suo Fei Ya	0.263	Income	−0.378**
		Education level	−0.145**
Jin An	0.377	Income	−0.350**
		Education level	−0.202**
Hui Zhan	Income	0.253	−0.331**
	Education level	0.253	−0.254**

The table shows multiple regression analysis *R*_adj_^2^ with standardised coefficient between income or education level and evaluation of acoustic comfort, where the significance levels (2-tailed) are also shown, with ^**^ indicating *p*<0.001.

It is noted that based on the data in Table 2 and Table 5, it can be shown that the space type of shopping malls, namely single- or multiple- space type, have no significant effect on users' evaluation of acoustics.

## Conclusions

Based on questionnaire surveys and measurements conducted in six shopping malls in Harbin City, China, this study examines the sound environment in terms of the users' social and behavioural characteristics.

In terms of social characteristics, evaluation of subjective loudness is influenced by income and occupation, with correlation coefficients or contingency coefficients of 0.10 to 0.40. The evaluation of acoustic comfort is influenced by income and education level, with correlation coefficients or contingency coefficients of 0.10 to 0.60. The effect of gender and age on the evaluation of subjective loudness and acoustic comfort is statistically insignificant.

In terms of behavioural characteristics, evaluation of subjective loudness is influenced by the reason for visit, frequency of visit, and length of stay, with correlation coefficients or contingency coefficients of 0.10 to 0.40. evaluation of acoustic comfort is influenced by the reason for visit, frequency of visit, length of stay, and season, with correlation coefficients of 0.10 to 0.30. The users who were waiting for someone were found to give lower evaluation of acoustic comfort compared those who were shopping; the users who went to shopping malls more than once a month were found to have higher evaluation of acoustic comfort; and the users who stayed in shopping malls from 2 to 4 hours were likely to give better evaluation of acoustic comfort evaluation compared to those who stayed longer or shorter.

Between different space types of shopping malls it seems that there is no significant difference in terms of acoustic evaluation.

The findings of this study can contribute to a better understanding of acoustic environment in shopping malls, and are also useful for the establishment of acoustic comfort prediction models based on artificial neural networks (ANN) and support vector machine (SVM), which are currently being developed. It is also of great interest to compare different kinds of shopping malls, using the same methodology. Correspondingly, a cross-cultural comparison between the UK and China is planned [53]. Finally, it is important to relate the evaluation of acoustic comfort to the sound fields of shopping malls, where the space forms are often special such as long or flat spaces, for which much theoretical work has been carried out [54]–[60].
